# Cognitive function in adolescence and the risk for premature diabetes and cardiovascular mortality in adulthood

**DOI:** 10.1186/s12933-018-0798-5

**Published:** 2018-12-05

**Authors:** Gilad Twig, Amir Tirosh, Estela Derazne, Ziona Haklai, Nehama Goldberger, Arnon Afek, Hertzel C. Gerstein, Jeremy D. Kark, Tali Cukierman-Yaffe

**Affiliations:** 1 Department of Medicine, Sheba Medical Center, Tel Hashomer, Ramat Gan, Israel; 2 The Israel Defense Forces Medical Corps, Ramat Gan, Israel; 3 The Dr. Pinchas Bornstein Talpiot Medical Leadership Program, Sheba Medical Center, Ramat Gan, Israel; 4 The Sackler School of Medicine, Tel Aviv University, Tel Aviv, Israel; 5 Institute of Endocrinology, Sheba Medical Center, Tel Hashomer, Ramat Gan, Israel; 6 Division of Endocrinology & Metabolism, and Population Health Research Institute, McMaster University & Hamilton Health Sciences, Hamilton, ON Canada; 7 The Division of Endocrinology, Diabetes and Hypertension, Brigham and Women’s Hospital, Harvard Medical School, Boston, MA USA; 8 Israel Ministry of Health, Jerusalem, Israel; 9 Hebrew University-Hadassah School of Public Health and Community Medicine, Ein Kerem, Jerusalem, Israel; 10 Department of Military Medicine, Hebrew University, Jerusalem, Israel; 11 Department of Epidemiology, Sackler School of Medicine, Herczeg institute on Aging, Tel-Aviv university, Tel-Aviv, Israel

**Keywords:** Adolescence, Cognitive performance, Diabetes, Mortality, Israelis

## Abstract

**Background:**

Epidemiological studies have demonstrated a relationship between cognitive function in youth and the future risk of death. Less is known regarding the relationship with diabetes related death. This study assessed the relationship between cognitive function in late adolescence and the risk for diabetes, cardiovascular- (CVD) and all-cause mortality in adulthood.

**Methods:**

This retrospective study linked data from 2,277,188 16–19 year olds who had general intelligence tests (GIT) conducted during pre-military recruitment assessment with cause of death as coded by the Israel Central Bureau of Statistics. The associations between cognitive function and cause-specific mortality were assessed using Cox models.

**Results:**

There were 31,268 deaths that were recorded during 41,916,603 person-years of follow-up, with a median follow-up of 19.2 (IQR 10.7, 29.5) years. 3068, 1443, 514 and 457 deaths were attributed to CVD, CHD, stroke, and diabetes, respectively. Individuals in the lowest GIT vs. highest GIT quintiles in unadjusted models had the highest risk for all-cause mortality (HR 1.84, 95% CI 1.78, 1.91), total CVD (HR 3.32, 95% CI 2.93, 3.75), CHD (HR 3.49 95% CI 2.92, 4.18), stroke (HR 3.96 95% CI 2.85, 5.5) and diabetes-related (HR 6.96 95% CI 4.68, 10.36) mortality. These HRs were attenuated following adjustment for age, sex, birth year, body-mass index, residential socioeconomic status, education and country of origin for all-cause (HR 1.23, 95% CI 1.17, 1.28), CVD (HR 1.76, 95% CI 1.52, 2.04), CHD (HR 1.7 95% CI 1.37, 2.11), stroke (HR 2.03, 95% CI 1.39, 2.98) and diabetes-related (HR 3.14 95% CI 2.00, 4.94) mortality. Results persisted in a sensitivity analyses limited to participants with unimpaired health at baseline and that accounted competing risk.

**Conclusions:**

This analysis of over 2 million demonstrates a strong relationship between cognitive function at youth and the risk for diabetes, all-cause and CVD-related mortality independent of adolescent obesity.

**Electronic supplementary material:**

The online version of this article (10.1186/s12933-018-0798-5) contains supplementary material, which is available to authorized users.

## Introduction

Epidemiological studies have demonstrated a relationship between cognitive function in youth and the future risk of death [1–3]. Such studies have shown that intelligence quotient (IQ) measured in childhood is positively associated with age of death and cardiovascular death in adulthood [4–7]. Childhood IQ has also been shown to be associated with chronic conditions such as hypertension and coronary heart disease [8–11]. In older adults, studies have reported a relationship between adult cognitive function and the risk of death [12], as well as with the incidence of cardiovascular disease (CVD) [13, 14] and diabetes [15]. Less is known regarding the relationship between cognitive function in adolescence (as measured by tools that discriminate between cognitively intact individuals) and the subsequent risk for diabetes-related death.

An unusual opportunity exists in Israel to assess the potential link between cognitive function in adolescence and risk for diabetes and cardiovascular-related mortality, as all eligible citizens are required by law to undergo a pre-military examination that includes both medical and psycho-social assessments. Thus extensive neuropsychological evaluation data and sociodemographic information is routinely collected in addition to a detailed medical examination. Linkage of this information with the Israel Central Bureau of Statistics officially coded cause of death database enables assessment of the relationship between cognitive function in late adolescence and the risk for diabetes and CVD-related death in adulthood.

## Materials and methods

This historical prospective cohort study linked data from a large unselected population who had pre-military health examination during late adolescence with their underlying cause of death as routinely coded by the Israel Central Bureau of Statistics. The relationships between cognitive function and all-cause mortality, CVD mortality, and CHD-, stroke- and diabetes-related mortality were assessed.

### Study population

One year prior to mandatory military service, at the age of 16–19 years (mean age 17.3 ± 0.4 years), all eligible Israeli adolescents are required to undergo medical and psychosocial assessment. Arab citizens, Druze women and orthodox/ultraorthodox religious Jewish women are exempt from military service and largely do not undergo this compulsory assessment. Information regarding country of origin, education and residential socioeconomic status (SES) is collected in addition to an extensive neuropsychological/cognitive assessment. This analysis pertains to 2,277,188 individuals who underwent pre-recruitment evaluation between the years 1967 and 2010, irrespective of whether they served or not. Excluded from this analysis are 81,213 individuals for whom cognitive data or BMI was unavailable, 92,301 members of non-Jewish minorities that were largely unrepresentative of their source populations and 3991 deaths that occurred from 1967 to 1980 for which the cause of death was not available. Of these, 3188 deaths were attributed to causes related to military service, and a simulation indicated that only 19 cardiovascular deaths were expected to be missed from analysis due to their young age [16, 17], thereby suggesting a negligible possible effect on the results.

### Evaluation of cognitive function at baseline

The general intelligence test (GIT), conducted as part of the pre-military recruitment assessment, has been used extensively as an investigative tool, as previously described [15, 18, 19]. It includes evaluation of language ability and intellectual performance, and comprises four sub-tests: the Otis-R which is a measure of the ability to understand and carry out verbal instructions; Similarities-R which assesses verbal abstraction and categorization; Arithmetic-R which assesses mathematical reasoning, concentration and concept manipulation; and Raven’s Progressive Matrices-R, which measures non-verbal abstract reasoning and visual-spatial problem solving abilities [20]. The sum of the scores of the 4 tests forms a validated measure of general intelligence (IQ) scored on a 9-point scale [21] that is adjusted from time to time. The GIT is administered by experienced personnel who undergo a 4-month training course.

### Mortality outcomes and documentation of cause of death

**Fig. 1:**
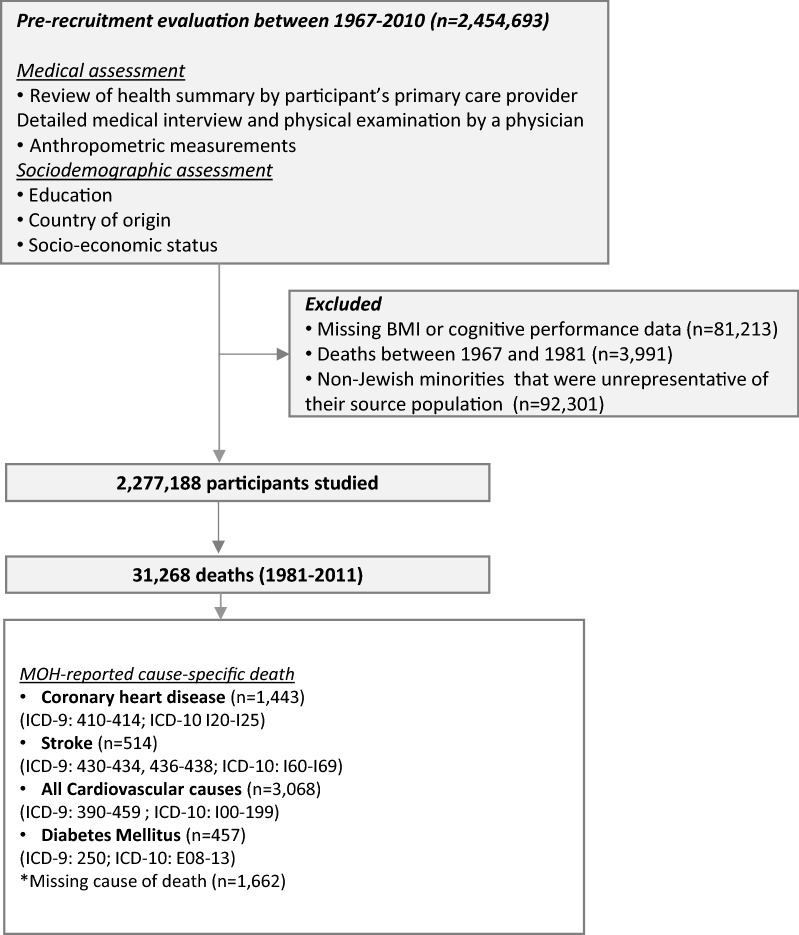
Flow diagram of study sample

Study outcomes were deaths that had occurred by June 30, 2011, officially coded from death notifications by the Israel Central Bureau of Statistics according to the International Classification of Disease (ICD) 9 revision (1981–1997) and ICD-10 revision (1998–2011). The official cause of death was unavailable before the year 1981. Deaths among Israel Defense Forces personnel have been computer-recorded since 1967 with a notation as to whether the death was service related. Outcomes analyzed were deaths attributed to all cardiovascular causes (ICD-9: 390–459; ICD-10: 100–99), to coronary heart disease (CHD) (ICD-9: 410–414; ICD-10: 120–125), stroke (ICD-9: 430–434, 436–438; ICD-10: 160–169) and diabetes (all types; ICD-9: 250; ICD-10: E08-13) as the underlying cause of death (see Fig. 1). Additionally, information regarding deaths from non-cardiovascular causes and all causes was also collected.

### Covariates

Weight and height were measured at baseline by trained medics [22]. BMI was calculated as weight (kg) divided by the squared height (m^2^). A general physical examination was conducted by military physicians who also reviewed the participants’ medical records and recorded standard diagnostic codes when relevant [23, 24]. Additional data regarding country of origin, education (divided into 4 categories: less than 9 years, 10 years, 11 years or 12 years) and residential SES were collected as detailed elsewhere [15, 25]. Place of origin was defined as the birthplace of the father or grandfather (if the father was born in Israel) and categorized according to country of origin [26, 27].

### Statistical analysis

Continuous variables were summarized using means and SD, and counts with percentages were used for binary variables. The GIT score was converted into annual sex-specific z scores based on all those that underwent GIT assessment in each specific calendar year and was grouped into five groups using quintiles. The distribution of baseline variables across the 5 groups was computed. Cox proportional hazard models were used to estimate the hazard ratios (HR) and 95% confidence intervals (CI) for time to first event for all cardiovascular mortality, for coronary heart disease (CHD), stroke, diabetes, non-cardiovascular-non-diabetes-related mortality and all-cause mortality comparing the highest quintile to the lower quintiles as well as for a unit change in the GIT z-score. The unadjusted analyses were repeated after adjustment for age, birth year and sex (Model 1); these and BMI (Model 2); Model 2 variables and residential SES (Model 3); and Model 3 variables and education and country of origin (Model 4). The analysis were also repeated after (a) accounting for the competing risk of death from other causes [28] (b) including only those individuals who were classified at their pre-military assessment to be of unimpaired health (i.e. no indication of any medical diagnosis in the pre-recruitment medical evaluation that would limit combat service) to minimize residual confounding as reported previously [16, 17, 27, 29, 30]. Analyses were performed with SPSS statistical software, version 23.0.

## Results

Between the years 1981 to 2011, 31,268 deaths were recorded during 41,916,603 person-years of follow-up, with a median follow-up of 19.2 (IQR 10.7, 29.5) years. Of these, 3068 deaths were attributed to all cardiovascular causes (CVD), 1443 to CHD, 514 to stroke, and 457 to diabetes (Fig. 1).

### Baseline characteristics

Table 1 depicts the baseline characteristics of the cohort distributed according to quintiles of GIT. Individuals in the highest quintile compared to the lowest quintile were more likely to have completed high school education, to be designated as having unimpaired health, to be taller, and to be of European or former USSR origin but less likely to be of Asian or African origin. They were also more likely to reside in a higher SES locality, and less likely to be either overweight or underweight.

**Table 1:** Baseline characteristics of the study participants according to quintiles of general intelligence test scores

	Q1	Q2	Q3	Q4	Q5	Total	p value for linear trend
N of participants	445,492	480,543	439,974	471,624	439,555	2,277,188	
Female %	31	43	44	44	40	40	
Age ± SD	17.4 ± 0.5	17.3 ± 0.4	17.3 ± 0.4	17.3 ± 0.4	17.3 ± 0.4	17.3 ± 0.4	0.158
BMI ± SD (kg/m^2^)	21.7 ± 3.6	21.7 ± 3.4	21.7 ± 3.3	21.6 ± 3.2	21.6 ± 3.1	21.6 ± 3.4	0.071
Underweight %	8.7	6.9	6.0	5.7	5.4	6.5	0.024
Overweight %	8.9	8.8	8.6	8.4	7.8	8.5	0.016
Obese %	4.9	4.0	3.5	3.3	3.0	3.7	0.011
Height ± SD (males)	171.8 ± 6.8	173.1 ± 6.8	173.6 ± 6.7	174.4 ± 6.7	175.1 ± 6.7	173.6 ± 6.8	0.001
Height ± SD (females)	160.9 ± 6.2	161.7 ± 6.1	162.0 ± 6.1	162.6 ± 6.0	163.1 ± 6.0	162.1 ± 6.1	0.001
Completed high school education (%)	56.9	76.3	82.1	91.0	96.0	80.5	< 0.001
Low SES %	33	26	23	20	19	24	0.001
Unimpaired health	77.6	80.3	81.9	82.6	80.3	81.2	0.069
Country of origin (%)							
Israel	4.6	5.0	5.7	6.4	7.6	5.8	0.003
USSR	9.0	10.9	11.8	14.7	16.1	12.5	0.001
Asia	30.6	29.5	26.6	21.9	16.0	25.0	0.007
Africa	38.1	30.8	24.5	17.8	11.8	24.7	< 0.001
Europe	13.7	22.6	30.9	39.0	48.4	30.8	< 0.001
Ethiopia	4.0	1.3	0.5	0.2	0.1	1.2	0.057*
Systolic/diastolic BP ± SD (mmHg)	116.5 ± 12.2/71.8 ± 8.3	115.9 ± 12.2/71.8 ± 8.2	116.3 ± 12.3/71.6 ± 8.2	116.3 ± 12.2/71.7 ± 8.2	117.1 ± 12.3/71.7 ± 8.2	116.4 ± 12.2/71.7 ± 8.2	0.110/0.319
Follow-up (mean ± SD) (years)	19.0 ± 9.8	19.2 ± 8.9	20.0 ± 10.2	19.0 ± 9.7	18.9 ± 10.3	19.2 ± 9.8	0.821
Median follow-up (25th; 75th) (years)	18.8 (10.3, 28.7)	18.9 (13.2, 27.0)	19.7 (10.6, 32.0)	18.9 (11.0, 29.3)	19.9 (9.3, 30.1)	19.2 (10.7, 29.5)	0.759
Cumulative follow-up (person-years)	8,064,856	8,840,363	8,453,920	8,577,783	7,979,682	41,916,603	0.414
Age at end of follow-up (± SD)	37.0 ± 12.1	37.0 ± 11.0	38.4 ± 12.7	36.9 ± 11.9	36.9 ± 12.5	37.2 ± 12.0	0.907

* Exponential trend p < 0.001

### GIT score and both all-cause and cause-specific mortality

Table 2a shows the distribution of mortality and its person-years incidence by cause across GIT quintiles. As can be seen, individuals in the lowest GIT quintile had the highest total mortality with a graded decrease towards the higher GIS quintiles. CVD-, CHD-, stroke-, diabetes- and non-CVD/non-diabetes-related mortality exhibited similar patterns.

**Table 2:** The association between fifths of GIT scores and cause-specific mortality

Mortality cause	Q1	Q2	Q3	Q4	Q5	Total
a. *Cause-specific death according to GIT quintiles*						
All-cause, N (%)	8765 (1.97%)	6306 (1.31%)	6188 (1.41%)	5221 (1.11%)	4788 (1.09%)	31,268
Incidence (event/10^5^ person-years)	108.7	71.3	73.2	60.9	60.0	74.6
Total cardiovascular, N (%)	1075 (0.24%)	641 (0.13%)	624 (0.14%)	394 (0.08%)	334 (0.08%)	3068
Incidence (event/10^5^ person-years)	13.3	7.25	7.4	4.6	4.2	7.3
Coronary heart disease, N (%)	526 (0.12%)	288 (0.06%)	308 (0.07%)	165 (0.03%)	156 (0.04%)	1443
Incidence (event/10^5^ person-years)	6.5	3.3	3.6	1.9	1.9	3.4
Stroke, N (%)	172 (0.04%)	115 (0.02%)	110 (0.03%)	72 (0.02%)	45 (0.01%)	514
Incidence (event/10^5^ person-years)	2.1	1.3	1.3	0.8	0.6	1.2
Diabetes, N (%)	184 (0.04%)	98 (0.02%)	96 (0.02%)	51 (0.01%)	28 (0.01%)	457
Incidence (event/10^5^ person-years)	2.3	1.1	1.1	0.6	0.4	1.1
Non-CVD, non-diabetes, N (%)	7506 (1.68%)	5567 (1.16%)	5468 (1.24%)	4776 (1.01%)	4426 (1.01%)	27,743
Incidence (event/10^5^ person-years)	93.1	63.0	64.7	55.7	55.4	66.2
b. *Cox proportional hazard models*						
All cause (unadjusted)	1.84 (1.78, 1.91)	1.25 (1.20, 1.29)	1.21 (1.16, 1.25)	1.04 (1.00, 1.08)	1	4.10*10^−231^
All cause (adjusted)	1.23 (1.17, 1.28)	1.08 (1.04, 1.13)	1.04 (1.00, 1.08)	1.01 (0.98, 1.06)	1	1.3*10^−33^
Total CVD (unadjusted)	3.32 (2.93, 3.75)	1.92 (1.68, 2.19)	1.71 (1.49, 1.95)	1.15 (0.99, 1.33)	1	2.13*10^−126^
Total CVD (adjusted)	1.76 (1.52, 2.04)	1.44 (1.24, 1.66)	1.27 (1.11, 1.46)	1.05 (0.91, 1.22)	1	1.3*10^−14^
CHD (unadjusted)	3.49 (2.92, 4.18)	1.87 (1.54, 2.27)	1.8 (1.48, 2.18)	1.03 (0.83, 1.29)	1	1.21*10^−66^
CHD (adjusted)	1.70 (1.37, 2.11)	1.36 (1.09, 1.68)	1.31 (1.07, 1.60)	0.95 (0.76, 1.18)	1	1.6*10^−9^
Stroke (unadjusted)	3.96 (2.85, 5.50)	2.60 (1.84, 3.66)	2.22 (1.57, 3.15)	1.56 (1.08, 2.27)	1	5.06*10^−24^
Stroke (adjusted)	2.03 (1.39, 2.98)	1.83 (1.26, 2.66)	1.61 (1.12, 2.30)	1.40 (0.96, 2.03)	1	3.3*10^−6^
Diabetes (unadjusted)	6.96 (4.68, 10.36)	3.76 (2.47, 5.72)	3.08 (2.02, 4.69)	1.81 (1.14, 2.87)	1	5.13*10^−38^
Diabetes (adjusted)	3.14 (2.00, 4.94)	2.36 (1.5, 3.7)	2.04 (1.32, 3.15)	1.55 (0.97, 2.46)	1	3.2*10^−9^
Non CVD/diabetes (unadjusted)	1.70 (1.64, 1.77)	1.18 (1.13, 1.23)	1.16 (1.11, 1.20)	1.02 (0.98, 1.06)	1	7.78*10^−247^
Non CVD/diabetes (adjusted)	1.17 (1.12, 1.22)	1.04 (1.00, 1.09)	1.01 (0.97, 1.05)	1.01 (0.97, 1.05)	1	1.3*10^−19^

The association between fifths of GIT scores (in comparison to highest quintile) and cause of death was determined using unadjusted and after adjustment for age, sex, birth year, body mass index (BMI), residential socioeconomic status, education and country of origin

*HR* Hazard ratio, *CI* confidence interval, *CHD* coronary heart disease, *CVD* cardiovascular

### Multivariable-adjusted relationship between GIT score and both all-cause and cause-specific mortality

**Fig. 2:**
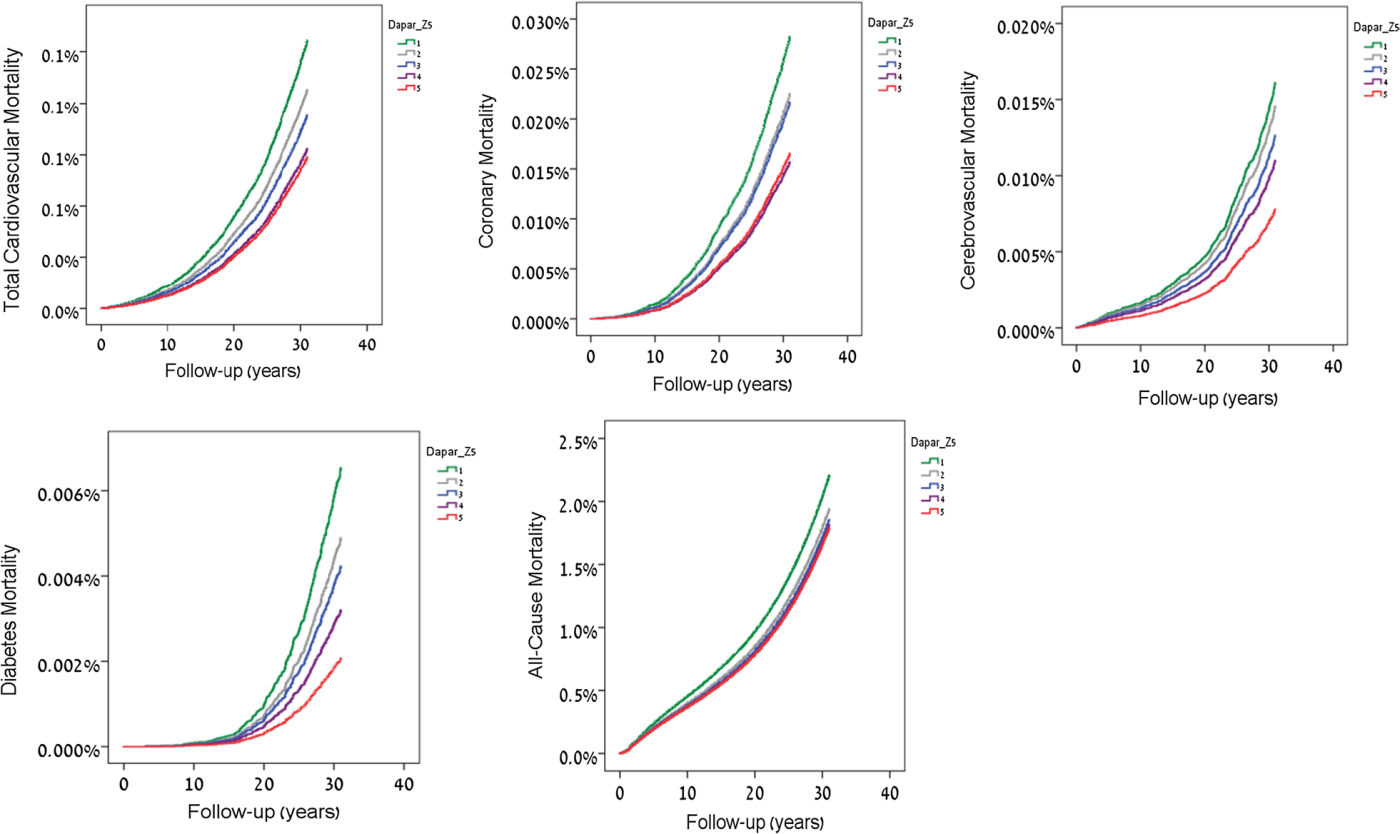
Cox hazard ratios for mortality due to all cardiovascular disease, coronary heart disease, stroke, diabetes, and all-cause mortality for the highest GIT quintile in comparison to lower quintiles after multivariable adjustment for age, sex, birth year, BMI, residential SES, education and country of origin (Model 4)

**Fig. 3:**
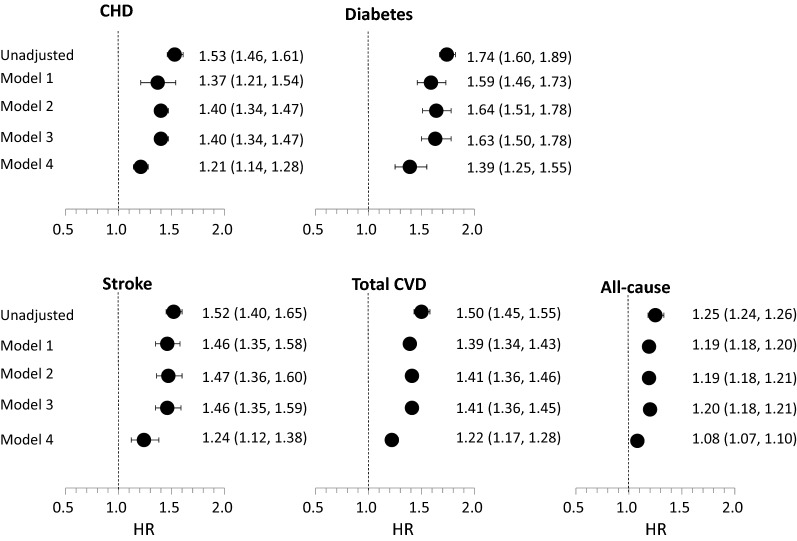
Cox hazard ratios for the relationship between a 1 unit lower GIT z-score on an interval scale and cause-specific mortality (all cardiovascular disease, CHD, stroke, diabetes, and all-cause mortality) in sequentially-adjusted models: age, birth year and sex (Model 1); these and BMI (Model 2); these and residential SES (Model 3); these and education and country of origin (Model 4)

The hazard ratios for total mortality and mortality due to cardiovascular disease, CHD, stroke and diabetes for the highest GIT quintile in comparison to other quintiles without adjustment and after adjustment for age, sex, birth year, BMI, SES, education and country of origin are presented in Fig. 2 and Table 2b. Overall, the lower the GIT the higher the hazard or all-cause and cause-specific mortality. As evident in Table 2b, individuals in the lowest GIT quintile vs. highest GIT quintile in the unadjusted models had the highest risk for all-cause mortality (HR 1.84, 95% CI 1.78, 1.91), and total CVD (HR 3.32, 95% CI 2.93, 3.75), CHD (HR 3.49 95% CI 2.92, 4.18), stroke (HR 3.96 95% CI 2.85, 5.5) and diabetes-related (HR 6.96 95% CI 4.68, 10.36) mortality. These HRs were attenuated following adjustment for Model 4 variables for all-cause mortality (HR 1.23, 95% CI 1.17, 1.28), total CVD (HR 1.76, 95% CI 1.52, 2.04), CHD (HR 1.7 95% CI 1.37, 2.11), stroke (HR 2.03, 95% CI 1.39, 2.98) and diabetes-related (HR 3.14 95% CI 2.00, 4.94) mortality, i.e. reduced by 72.6%, 67.2%, 71.9%, 65.2%, and 64.1%, respectively. Figure 3 depicts the relationship between a 1 unit lower GIT z-score on an interval scale and the risk for cause-specific mortality. As can be seen, serial adjustments for the various covariates attenuated the relationship modestly in Models 1, 2 and 3, but strongly in Model 4 which also adjusted for education, residential SES and origin, although the associations remained statistically significant. An additional analysis accounting for the competing risk of death from other causes yielded similar results (Additional file 1: Table S1). A sensitivity analysis that included only those individuals with unimpaired health at baseline (N = 1,656,827) tended to attenuate the associations which, however, remained graded across quintiles of GIT and statistically significant (Additional file 1: Table S2).

## Discussion

This analysis of 2,277,188 adolescents followed for a median of 19.2 years demonstrates an inverse association between cognitive scores at the age of ~ 17 years and the risk for all-cause death, cardiovascular mortality, and CHD, stroke and diabetes-related death. The strongest relationship observed was for the cognitive function and diabetes mortality. For example, there was a ~ 1.8-fold greater multivariable-adjusted hazard for total cardiovascular disease mortality in those who were in the lowest vs. the highest GIT score category, while for diabetes-related death a ~ threefold greater hazard was observed. The associations, although substantially diminished after serial adjustments for variables that are known to be associated with both cognitive function and mortality (such as SES, education, origin and BMI), remained statistically significant.

This analysis is supported by data from previous studies conducted in children and in older individuals. An analysis of ~ 31,000 individuals with previous CVD reported a relationship between the Mini Mental State Examination Score (MMSE, a screening instrument for dementia) and incident cardiovascular events after adjustment for demographic and cardiovascular risk factors [14]. In an analysis of 11,140 individuals with type 2 diabetes who were followed for a median of 5 years there was also an inverse relationship between the MMSE score at baseline and incident CVD events that persisted after multivariate adjustment for sociodemographic variables [31]. In a study of 9204 individuals participating in the English Longitudinal study of Ageing, cognition was inversely associated with death from cancer, cardiovascular disease, respiratory illness and other causes [32]. These association were attenuated after adjustment for demographic and SES variables but remained significant. There have been several studies in young adults. An inverse relationship between cognitive scores measured as part of military conscription in Denmark and risk for diabetes and for cardiovascular death was reported; the relationship was attenuated after adjustment for education but remained significant [12]. We have previously reported an inverse relationship between cognitive scores determined in adolescence and the subsequent risk of diabetes and dysglycemia [15, 33]. The findings of our study strengthen the results of these studies and further suggest that sensitive measures of cognitive function can discriminate between younger individuals with a higher/lower risk for diabetes and CVD related death.

There are several explanations for the relationship observed between cognitive function at age ~ 17 and the risk for diabetes and CVD-diabetes related death. First, as cognitive function is associated with education and SES, it may be that the relationship observed is a reflection of the already recognized relationship between these variables (and their possibly inadequate adjustment) and the subsequent risk for CVD death [34–38]. A limited number of socio-demographic variables were available for adjustment in our study; thus it may well be that unmeasured or insufficiently discriminating socio-demographic factors, especially in the early years of the study, that affect the GIT account for the relationship. The strong reduction in the hazard ratios on multivariable adjustment, particularly for education, residential SES and origin (ie., model 4 adjustment), supports this explanation. Education is an increasingly non-discriminating variable in our data set as rapidly growing proportions over time have 12 years of education. Furthermore, we used an ecological measure of SES—based on locality of residence, which lacks refinement in cities. Therefore, residual confounding cannot be excluded as an explanation for the associations we report in multivariable-adjusted analyses. Alternatively, differences in GIT might be associated with different life-style behavioral patterns in childhood or specific intra-utero exposures (also referred to as fetal origin of adult disease [FOAD]) [39]. For example it has been suggested that low birth weight, a surrogate marker of poor nutrition and fetal growth, is associated with stroke, coronary heart disease, stroke and diabetes [40, 41]. Additionally, it could be that physical activity and diet in childhood could have affected intelligence scores and the subsequent risk for diabetes and CVD death in an independent manner. The observation of a shorter stature among adolescents with lower GIT may argue in favor of a common mechanism for these observation. For example, poor nutrition in early life and perhaps already in-utero may independently affect neurocognitive development (and thus lower GIT) [42–44], growth velocity (thus shorter stature) [45, 46] and may increase the risk for future development of the metabolic syndrome [45, 47] and its associated cardio-metabolic risk. An additional possible explanation is that a higher GIT score leads to greater achievements, higher income, higher SES, a more healthy lifestyle, less exposure to a deleterious life-style, better access to healthcare and a better ability to prevent and manage disease [48–50]. Finally, it could be that the relationship observed suggests a common origin(s) or pathway for both cognitive function and dyglycemia. These might include, among others, mitochondrial (dys)function [51, 52], the sortilin pathway [53], activation of the hypothalamic-pituitary-adrenal axis, inflammation, dysglycemia *perse* or brain and systemic insulin sensitivity [54–56]. Supporting the latter two explanations is the relatively strong relationship observed between cognitive function and diabetes-related mortality.

The study has several limitations. First, only a limited number of potential confounders were available for adjustment. Thus, information related to socio-demographic variables was restricted to country of origin, education and a residential-based index of SES in adolescence. Information related to individual measures of SES and to life-style factors such as diet, physical activity and smoking in adolescence (and adulthood) were not available, thus limiting our ability to assess an independent relationship (or an association mediated through these risk factors). Second, data regarding CVD mortality were not available from 1967 to 1981, however most cases of death during those years were attributable to service-based trauma, whereas the expected number of CVD or diabetes deaths was trivial [16]. The study has several strengths including the large sample size and consequently substantial statistical power, the inclusion of data with respect to both women and men, the largely unselected population-based sampling and the ability to restrict the analysis to a healthy population.

## Conclusions

To conclude, this analysis demonstrates an inverse relationship between cognitive function at the age of 17 and the risk for diabetes-related death as well as all-cause mortality and CVD-related death. The study’s results emphasize the need for further research aimed at assessing an independent association of adolescent cognition with subsequent mortality as well as unravelling possible mechanistic routes that may explain the strong cognitive–diabetes relationship. On a clinical level, these results highlight the ability of cognitive tests to discriminate between individuals at higher and lower risk for CVD/diabetes death, thus possibly enabling targeted risk mitigation strategies and more intense follow-up for these high risk populations.

## Additional file

**Additional file 1.** Additional analyses supporting the association between the general intelligence test score and cause-specific mortality.

## Abbreviations

IQ: intelligence quotient
SES: socioeconomic status
GIT: general intelligence tests
CVD: cardiovascular
CHD: coronary heart disease
BMI: body mass index
IQR: inter-quartile range
