# Supplementary material for: Cognitive function in adolescence and the risk for premature diabetes and cardiovascular mortality in adulthood
Source: Cardiovasc Diabetol. 2018 Dec 5;17:154. doi: 10.1186/s12933-018-0798-5 (PMC6280532; doi:10.1186/s12933-018-0798-5)
Supplement: Supplementary file 1 — Additional file 1. Additional analyses supporting the association between the general intelligence test score and cause-specific mortality. [file 12933_2018_798_MOESM1_ESM.docx]

**Additional file 1**

**Appendix Table S1: The association between fifths of GIT scores (in comparison to highest fifth) and causes of death (unadjusted) accounting for the competing risk of other causes of death**

| **Mortality cause** | **F1**  **HR (95% CI)** | **F2**  **HR (95%CI)** | **F3**  **HR(95%CI)** | **F4**  **HR(95%CI)** | **F5**  **HR(95%CI)** |
| --- | --- | --- | --- | --- | --- |
| **Total CVD** | 3.29 (2.91, 3.72) | 1.91(1.67, 2.18) | 1.70(1.49, 1.95) | 1.15(0.99,1.33) | 1 |
| **CHD** | 3.45 (2.89, 4.14) | 1.86(1.53, 2.26) | 1.79(1.48, 2.17) | 1.03(0.83, 1.29) | 1 |
| **Stroke** | 3.92(2.83, 5.45) | 2.58(1.83, 3.65) | 2.22 (1.57, 3.14) | 1.56 (1.08, 2.27) | 1 |
| **Diabetes** | 6.88(4.62, 10.23) | 3.73(2.45, 5.69) | 3.07 (2.02, 4.68) | 1.81 (1.14, 2.87) | 1 |
| **Non CVD/ Non Diabetes** | 1.70 (1.64, 1.77) | 1.04 (1.13, .23) | 1.01 (1.11, 1.20) | 1.01(0.98, 1.06) | 1 |

F-fifths of GIT; HR- Hazard ratio; CI- confidence interval; CHD- Coronary Heart Disease;

**Appendix Table S2: The association between fifths of GIT scores (in comparison to highest fifth) and cause of death after adjustment for age, sex, birth year, body-mass index, residential socioeconomic status, education and country of origin (model 4) (Cox proportional hazards modeling) in a subset of individuals with unimpaired health at enrollment (N=1656827).**

| **Mortality cause** | **F1**  **HR (95% CI)** | **F2**  **HR (95%CI)** | **F3**  **HR(95%CI)** | **F4**  **HR(95%CI)** | **F5**  **HR(95%CI)** |
| --- | --- | --- | --- | --- | --- |
| **Total CVD** | 1.77(1.49, 2.19) | 1.44(1.22, 1.70) | 1.28(1.11, 1.46) | 1.05(0.92, 1.22) | 1 |
| **CHD** | 1.60 (1.25, 2.05) | 1.40 (1.1, 1.78) | 1.30 (1.03, 1.63) | 0.96(0.75, 1.24) | 1 |
| **Stroke** | 1.87(1.23, 2.86) | 1.52(1.00, 2.31) | 1.47(0.99, 2.18) | 1.16(0.76, 1.76) | 1 |
| **Diabetes** | 2.83 (1.67, 4.81) | 1.99(1.17, 3.39) | 1.75(1.05, 2.92) | 1.61(0.95, 2.75) | 1 |
| **Non CVD/Non Diabetes** | 1.13(1.07, 1.19) | 1.02(0.97, 1.07) | 1.00(0.96, 1.05) | 0.99(0.94, 1.04) | 1 |
